# Predictors of antimicrobial use in intensive care unit patients

**DOI:** 10.1017/ash.2023.269

**Published:** 2023-09-29

**Authors:** Owen Albin, Jonathan Troost, Keith Kaye

## Abstract

**Background:** Identification of predictors of antibiotic use can inform targeted antimicrobial stewardship initiatives and can account for sources of bias in before-and-after interventional stewardship studies. To date, no study has identified clinical predictors of antimicrobial use within intensive care units (ICUs), where antimicrobial resistance is most prevalent and problematic. **Methods:** As part of an ongoing prospective, single-arm, pilot feasibility trial of an ICU diagnostic stewardship intervention, we performed a nested retrospective cohort study to explore associations between patient clinical variables and ICUs antimicrobial use and resistance rates (AURs). We included all patients hospitalized in 3 ICUs (surgical, medical, and cardiac) from 2017 to 2021 at Michigan Medicine, a large, tertiary-care, academic medical center. Data were extracted from the electronic medical record using a structured query. Admission-level data were captured, including patient demographics, medical comorbidities, *International Classification of Disease, Tenth Revision* (ICD-10) admission diagnoses, as well as calendar day-level data including vital signs, clinical and microbiologic laboratory data, measures of acute severity of illness, ventilator–supplemental oxygen metrics, and procedural interventions using current procedural terminology (CPT) codes. ICU AURs were defined as total antibiotic days of therapy per patient per 100 ICU days. Associations between clinical variables and ICU AURs were calculated as rate ratios (RRs). Multiple imputation using fully conditional specification was performed to create 25 imputation data sets. Negative binomial regression models were constructed for each data set using backward selection. Variables retained in >50% of models were included in a final multivariate model. **Results:** In total, 15,177 ICU patient admissions were captured. Age, sex assigned at birth, and race did not independently associate with ICU AURs. Comorbidities, medical interventions, admission diagnoses, and laboratory data that independently associated with ICU-AURs are shown in Table 1. The clinical variables most strongly associated with increased ICU-AURs were pneumonia (RR, 1.55; 95% CI, 1.451.64), bacteremia (RR, 1.35; 95% CI, 1.25– 1.46), intraabdominal infection (RR, 1.35; 95% CI, 1.18–1.55), SOFA score (RR, 1.27; 95% CI, 1.14–1.42), abnormal WBC (RR, 1.26; 95% CI, 1.20–1.32), and immunocompromised status (RR, 1.20; 95% CI, 1.10–1.31). Clinical variables most strongly associated with decreased ICU-AURs were cardiac ICU (RR, 0.56; 95% CI, 0.52–0.60), COVID-19 (RR, 0.62; 95% CI, 0.56–0.70), and receipt of an invasive nonsurgical procedure (RR, 0.90; 95% CI, 0.82–0.98). **Conclusions:** In this single-center retrospective cohort study, several clinical variables were independently associated with ICU-AURs. These results may be used to identify patient subgroups for potentially high-yield ICU-based stewardship interventions and to account for sources of bias in before-and-after studies of ICU-based stewardship interventions.

**Figure:**
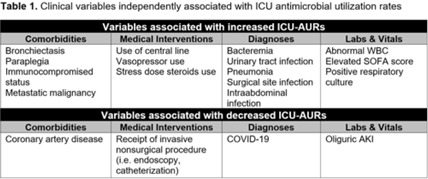

**Disclosures:** None

